# Are seroindeterminate western blot patterns in human T-Cell lymphotropic virus type 1 (Htlv-1) infected individuals associated with low proviral load levels?

**DOI:** 10.1186/1742-4690-12-S1-P63

**Published:** 2015-08-28

**Authors:** Camila Cánepa, Jimena Salido, Sindy Fraile, Matías Ruggieri, Mirna Biglione, Carolina Berini

**Affiliations:** 1 Instituto de Investigaciones Biomédicas en Retrovirus y SIDA (INBIRS), UBA-CONICET, Buenos Aires, Argentina

## 

For HTLV-1/2 diagnosis, reactive screening results are generally confirmed by Western Blot (WB). However, the large number of indeterminate WB patterns is still a problem worldwide. The aim of this study was to determine whether low levels of proviral load (PVL) are associated with seroindeterminate results by WB. PVL was determined in 49 HTLV-1 samples confirmed by n-PCR and classified as G1: positive by WB from individuals with disease (n= 27, 5 ATLL and 22 HAM/TSP); G2: positive by WB from asymptomatic carriers (n= 18); and G3: seroindeterminate samples by WB from asymptomatic carriers (n= 4). The viral gen pol and albumin were quantified by real-time SYBR Green PCR (ABI Prism System-AppliedBiosystems 75). Calibration curves were constructed using a DNA stock from MT2 cell line (Limit of quantification: 3 pol copies/reaction-R2> 0.99) and the analysis was performed by Krustall Wallis (GraphPad Prism v.5). Median PVL values were 4.03, 1.58 and 0.15 copies of HTLV-1/1 PBMCs fo r G1, G2 and G3, respectively. Correlation between PVL and age of patients (S= 0.61) was found for G1 samples. PVL median values were significantly different between the three groups (p= 0.3); the difference was also observed (p = 0.5) when considering HTLV-1 positive samples by WB [G1 + G2] as a single group. Even though a seroconversion could not be discarded in seroindeterminate cases, a low viral replication rate due to other factors could trigger a weak immune response, thus causing seroindeterminate WB patterns. The present study clearly demonstrates that such cases could be associated to a low HTLV-1 PVL and that the molecular analysis is a necessary tool to reach a final diagnosis.

